# Supplementary material for: Content-rich biological network constructed by mining PubMed abstracts
Source: BMC Bioinformatics. 2004 Oct 8;5:147. doi: 10.1186/1471-2105-5-147 (PMC528731; doi:10.1186/1471-2105-5-147)
Supplement: Additional File 2 — The original results of the above study (non-essential files are deleted to keep the file size under the limit set by BMC bioinformatics). [file 1471-2105-5-147-S2.bz2 › chilibotAdditionalFile2/dip05/235ID8006584E759/html/IL2RB_IL2.html]

 


 **IL2RB** and **IL2** 
  
Found 3819 abstracts in PubMed, retrieved 05.  
 

 What does Google say? 
 PDF only 
| .edu only 

---

**Interactive relationship** (e.g. stimulation, inhibition, etc)

**Non-interactive relationship** (e.g. studied together, co-existance, homology, etc.)

- Plasticity of IL 2  [ **IL2** ]  and IL 2  [ **IL2** ]  receptor  [ **IL2RB** ]  chains in rat lymphoid tissues in situ after stimulation with staphylococcal enterotoxin A.  Ref: 12890448 Cytokine, 2003
- The pathological changes of the graft, IL 2  [ **IL2** ]  receptor  [ **IL2RB** ]  expression in lamina propria lymphocytes, serum IL 2  [ **IL2** ]  concentrations, results of spleen lymphocytes transformation test and the IL 2  [ **IL2** ]  secretion capacity were determined and compared.  Ref: 12875685 Chin Med J (Engl), 2003
- Immunosuppression protocol anti IL 2  [ **IL2** ]  receptor  [ **IL2RB** ]  antibodies, steroids, mycophenolate mofetil MMF.  Ref: 12876872 Acta Med Croatica, 2003
- Addition of IL 12 to this coculture prevented apoptosis of the CD4 T cells, which was associated with up modulation of IL 2  [ **IL2** ]  receptor  [ **IL2RB** ]  beta chain expression.  Ref: 12883484 Hepatology, 2003
- The suppressive activity of CD8 CD25 thymocytes was abrogated by a mixture of anti CTLA 4 and anti TGF beta1 antibodies and it was mediated by their ability to inhibit the expression of the IL 2  [ **IL2** ]  receptor  [ **IL2RB** ]  alpha chain on target T cells.  Ref: 12893750 BloodBlood, 2003
- Although the effects of mitogens on the synthesis of interleukin 2  [ **IL2** ]  IL 2  [ **IL2** ]  and IL 2  [ **IL2** ]  receptor  [ **IL2RB** ]  IL 2r have been described, a detailed in situ analysis of the spatio temporal changes of the expression of the IL 2  [ **IL2** ]  gene and the three IL 2r components in lymphoid tissues is still missing.  Ref: 12890448 Cytokine, 2003
